# A Highly-Sensitive Picric Acid Chemical Sensor Based on ZnO Nanopeanuts

**DOI:** 10.3390/ma10070795

**Published:** 2017-07-13

**Authors:** Ahmed A. Ibrahim, Preeti Tiwari, M. S. Al-Assiri, A. E. Al-Salami, Ahmad Umar, Rajesh Kumar, S. H. Kim, Z. A. Ansari, S. Baskoutas

**Affiliations:** 1 Department of Chemistry, Faculty of Science and Arts, Najran University, P.O. Box 1988, Najran 11001, Saudi Arabia; ahmedragal@yahoo.com (A.A.I.); semikim77@gmail.com (S.H.K.); 2 Promising Centre for Sensors and Electronic Devices (PCSED), Najran University, P.O.Box-1988, Najran 11001, Saudi Arabia; msassiri@gmail.com; 3 Department of Materials Science, University of Patras, Patras GR-26504, Greece; bask@upatras.gr; 4 Centre for Interdisciplinary Research in Basic Sciences, Jamia Millia Islamia, New Delhi 110025, India; preet.19.pt@gmail.com (P.T.); zaansari@jmi.ac.in (Z.A.A.); 5 Department of Physics, Faculty of Science and Arts, Najran University, P.O. Box 1988, Najran 11001, Saudi Arabia; 6 Department of Physics, Faculty of Science, King Khalid University, P.O. Box-9004, Abha 61413, Saudi Arabia; Salami11@gmail.com; 7 PG Department of Chemistry, JCDAV College, Dasuya, Punjab 144205, India; rk.ash2k7@gmail.com

**Keywords:** ZnO nanopeanuts, hydrothermal, electrochemical sensor, picric acid

## Abstract

Herein, we report a facile synthesis, characterization, and electrochemical sensing application of ZnO nanopeanuts synthesized by a simple aqueous solution process and characterized by various techniques in order to confirm the compositional, morphological, structural, crystalline phase, and optical properties of the synthesized material. The detailed characterizations revealed that the synthesized material possesses a peanut-shaped morphology, dense growth, and a wurtzite hexagonal phase along with good crystal and optical properties. Further, to ascertain the useful properties of the synthesized ZnO nanopeanut as an excellent electron mediator, electrochemical sensors were fabricated based on the form of a screen printed electrode (SPE). Electrochemical and current-voltage characteristics were studied for the determination of picric acid sensing characteristics. The electrochemical sensor fabricated based on the SPE technique exhibited a reproducible and reliable sensitivity of ~1.2 μA/mM (9.23 μA·mM^−1^·cm^−2^), a lower limit of detection at 7.8 µM, a regression coefficient (*R*^2^) of 0.94, and good linearity over the 0.0078 mM to 10.0 mM concentration range. In addition, the sensor response was also tested using simple *I-V* techniques, wherein a sensitivity of 493.64 μA·mM^−1^·cm^−2^, an experimental Limit of detection (LOD) of 0.125 mM, and a linear dynamic range (LDR) of 1.0 mM–5.0 mM were observed for the fabricated picric acid sensor.

## 1. Introduction

Electrochemical nanotechnology is an emerging combinational technique that involves electrochemical methods, and nanotechnology has been explored for many important applications in fields such as gas sensors, biosensors, electrochemical sensors, electronics, photovoltaic devices, supercapacitors, pH sensors, and humidity sensors [1,2]. Among the various applications, electrochemical detection and the sensing of hazardous and toxic chemicals are of utmost importance. Semiconductor metal oxide nanomaterials act as efficient electron mediators for the modification and fabrication of highly sensitive electrodes [3,4]. Among the various metal oxide nanomaterials, the ZnO–II-VI semiconductor, with a low band gap energy of 3.37 eV and a large exciton binding energy of 60 MeV, is extensively studied [5]. Due to its tetrahedral structure and polar symmetry along the hexagonal axis of the wurtzite phase, ZnO with a variety of morphologies having high surface defect density can be synthesized [6,7,8,9,10,11,12,13,14]. These morphologies provide a large surface area for the adsorption of chemical species, which is a key factor for efficient electrochemical sensor applications [11,15]. As ZnO nanomaterials are *n*-type semiconductors, the adsorbed chemical species are reduced. Such redox changes on the surface of ZnO nanomaterials make these materials efficient electron mediators in the electrochemical sensor fabrication process [10,13].

ZnO nanostructures of different morphologies have been recently reported in the literature for their electrochemical applications for the detection of harmful, toxic, and even biologically important chemical substances [16,17,18]. Molaakbari et al. [19] fabricated a carbon paste electrode modified with ZnO nanorods and 5-(4`-amino-3`-hydroxy-biphenyl-4-yl)-acrylic acid (3,4`-AAZCPE) electrochemical sensors for levodopa, a precursor of the neurotransmitter dopamine, which is widely used in the clinical treatment of Parkinson’s disease. The selective and sensitive determination of calcitonin was also reported by Patra et al. [20] in human blood serum samples using an electrochemical sensor comprising a medullary thyroid carcinoma marker imprinted polymer onto the surface of ZnO nanostructures. High sensitivity, a low detection limit, and a response time of ~26.58 μA·cm^−2^·mM^−1^, ~5 nM and 10 s, respectively were observed during the electrochemical sensing of ammonia at room temperature using ZnO nano pencil based electrochemical sensors [21,22]. Mehta et al. [23] reported an ultra-high sensitivity of ~97.133 μA·cm^−2^·μM^−1^ and a very low detection limit of 147.54 nM for hydrazine using a well-crystallized ZnO nanoparticle based amperometric sensor. A pristine ZnO nanorods array deposited on an inert alloy substrate were used as an efficient electron mediator for the fabrication of a hydrazine electrochemical sensor with a sensitivity of ~4.48 μA·μM^−1^·cm^−2^ [24]. One-dimensional (1D) ZnO nanorods and two-dimensional (2D) ZnO nanoflakes synthesized on an Au-coated substrate through a sonochemical approach showed 11.86 and 7.74 μA·M^−1^ sensitivities, respectively, for cortisol, a steroid hormone [25]. Additionally, other chemicals such as glucose [26,27,28,29], urea [30,31], uric acid [32], ethanol [33,34,35,36], nitrophenols [37], trinitrotoluene [38], nitrophenyl amine [39,40], and ethanolamine [41] are also detected through ZnO nanostructure-mediated electrochemical sensors.

Phenols and their derivatives, particularly 2,4,6-Trinitrophenol (Picric acid), find extensive application in many industries, such as pharmaceuticals, polymers, leathers, agriculture, fuel cells, and explosives [10,14]. Picric acid is a highly toxic and carcinogenic chemical and drastically affects the liver, kidney, eyes, and the respiratory tract [42,43]. A fast, reliable, and selective detection and sensing of even a low level of picric acid is thus required.

In this report, we present a facile, low-temperature solution method for ZnO nanoparticles with peanut shapes. Further, a highly sensitive picric acid electrochemical sensor based on ZnO nanopeanuts was fabricated for the sensing of picric acid.

## 2. Experimental Details

### 2.1. Materials

For the synthesis of the ZnO nanopeanuts and the fabrication of the picric acid electrochemical sensor, Zinc nitrate hexahydrate (Zn(NO_3_)_2_·6H_2_O), polyethylene glycol, butyl carbitol acetate (BCA), and ammonium hydroxide (NH_4_OH) were purchased from Loba Chemie (Mumbai, India), and were used as received without any further purification. Triple deionized (DI) water was used as a solvent for the preparation of solutions.

### 2.2. Synthesis of ZnO Nanopeanuts

In a typical reaction process for the synthesis of ZnO nanopeanuts, 0.1 M Zinc nitrate hexahydrate (Zn(NO_3_)_2_·6H_2_O), was dissolved in 50 mL of DI water and mixed well, stirring with 0.2 g polyethylene glycol prepared in 50 mL of DI water. The stirring was continued for 30 min. After stirring, a few drops of ammonium hydroxide (NH_4_OH) were mixed in the resultant solution to maintain the solution at a pH = 10.15. The resultant solution was again stirred for 20 min, and then transferred to a Teflon-lined autoclave for hydrothermal reaction at 170 °C for 7 h. On completion, the autoclave was cooled to room temperature, the white precipitates were decanted and washed with DI water to neutralize the pH, and finally dried at 80 °C in a convection oven.

### 2.3. Characterizations of ZnO Nanopeanuts

Detailed analytical and characterization techniques were used for the evaluation of the morphological, structural, crystalline, and optical properties of the hydrothermally synthesized ZnO nanopeanuts. Field emission scanning electron microscopy (FESEM; JEOL-JSM-7600F, JEOL Ltd., Tokyo, Japan) attached with Energy Dispersive Spectroscopy (EDS) was used to study the morphological, structural, and compositional features of the ZnO nanomaterials. Phase crystallinity and microstructural parameters were evaluated through X-ray diffraction (XRD, PAN analytical Xpert Pro.) in the scan range of 10°–80° (2θ) angles using a Cu-Kα radiation source with a wavelength of 1.54 Å. The compositional, optical, and Raman scattering spectral properties of the ZnO nanopeanuts were analyzed through Fourier transform infrared spectroscopy (FTIR; Perkin Elmer-FTIR Spectrum-100, Perkin Elmer, Germany) in the scan range of 450–4000 cm^−1^, a UV-visible spectrophotometer (Perkin Elmer-UV/VIS-Lambda 950, Perkin Elmer, Germany) in the absorption range of 200–800 nm, and Raman scattering spectroscopy (Perkin Elmer-Raman Station 400 series, Perkin Elmer, Germany) in the scan range of 200–700 cm^−1^, respectively.

### 2.4. Fabrication of Electrochemical Sensor Based on Screen Printed Electrode (SPE)

**Scheme 1:**
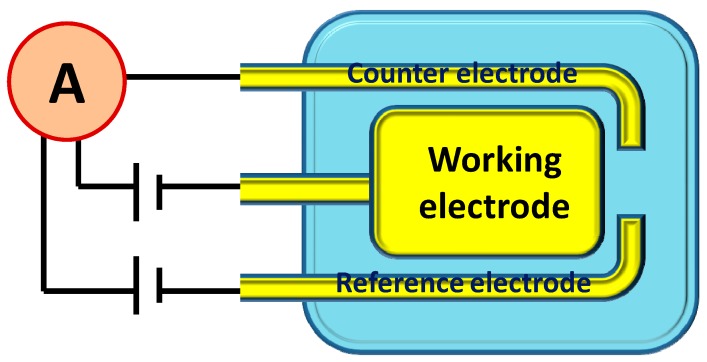
Schematic representation of a screen printed electrode (SPE).

Two sensor techniques i.e., electrochemical and simple current-voltage (*I-V*) techniques, were chosen to characterize the fabricated picric acid sensor using synthesized ZnO nanopeanuts. The in-house SPE was fabricated using Printed circuit board (PCB) technology on a glass epoxy substrate, which consisted of a three electrode system viz. working, counter, and reference electrode. All of the three electrodes were gold plated (Scheme 1). The working electrode (surface area 0.13 cm^2^) was used for the coating of synthesized ZnO nanopeanuts by formulating a thick paste as reported elsewhere [44]. The paste was prepared by mixing a known and optimized amount of BCA (30%) in nanomaterial (70%), then printing it on the SPE, and drying it at 80 °C. BCA is known as an organic binder, and when mixed in an optimized ratio of 30:70 (BCA:nanomaterial), it results in a good thixotropic paste. The SPE is expected to play the role of conducting electrons from analyte/ZnO to the potentiostat. The cyclic voltammogram (CV) of the SPE was obtained using IVIUM’s potentiostat/galvanostat at room-temperature. Different picric acid solutions of 0.0078 mM, 0.078 mM, 0.78 mM, 2 mM, 5 mM, and 10 mM were prepared in 0.1 M phosphate buffer solution (PBS) (pH = 7.4). The curves were obtained at a fix scan rate of 50 mV·s^−1^, while the potential was varied from −1.0 to 1.0 V. CV measurements were also performed at various scan rates (50 to 500 m·Vs^−1^) at one concentration of picric acid (2 mM).

### 2.5. Fabrication of Picric Acid Sensor Based on Current-Voltage Technique Measurements

For sensors based on the *I-V* characteristic, a different set of electrodes were prepared. A cleaned silver electrode (AgE, surface area = 0.0214 cm^2^, Purity supplier) was used as one of the electrodes (working), onto which a film of nanomaterial was coated using the paste prepared for the SPE’s coating. A platinum wire was used as a counter electrode. Before coating, the Ag electrodes were rubbed against an alumina gel, followed by ultrasonic cleaning and repeated washings with deionized water. The electrode was dried in an air oven for 6 h at 70–75 °C. A Keithley electrometer, 6517A (USA) was used for measuring the current–voltage parameters at room temperature conditions. A platinum wire was used a counter electrode. The picric acid solutions were prepared in 0.1 M phosphate buffer solution (PBS) having a pH = 7.4 in the scan range of 0.0–4.0 V.

## 3. Results and Discussion

### 3.1. Characterizations and Properties of ZnO Nanopeanuts

**Figure 1:**
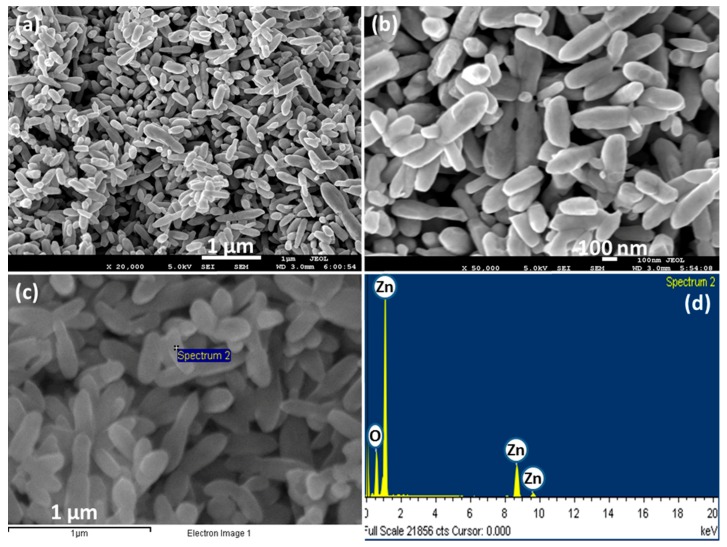
Typical (**a**,**b**) FESEM images; (**c**) EDS-SEM microscopic image and (**d**) EDS spectrum of the as-synthesized ZnO nanopeanuts.

The general morphologies of the synthesized material were examined by FESEM and the observed results are shown in Figure 1a,b. The observed FESEM images revealed that the prepared materials possess peanut shaped morphologies and grow in high density with almost uniform shape and size. The surface of the peanut shaped ZnO is highly rough, with swollen edges and a narrow central part. It is interesting to see that due to the high density growth, some nanopeanuts are linked to each other through one of their surfaces. The average diameter and length of ZnO nanopeanuts is ~110 ± 20 nm and ~220 ± 20 nm, respectively. Additionally, some dumb-bell and rod-shaped morphologies are also formed due to the aggregation of two ZnO nanopeanuts through their ends.

The rough surface of the ZnO peanuts provides a sufficiently large surface area for intermolecular π stacking of the electron deficient picric acid benzene ring, which further facilitates a charge transfer from *n*-type semiconducting ZnO to picric acid molecules. The electron deficient nature of the picric acid benzene ring can be accounted for due to the presence of electron withdrawing nitro (–NO_2_) groups. Additionally, the surface active sites of ZnO nanopeanuts attract the lone pairs of electrons present on the –OH group of the picric acid. The extent of chemisorptions is therefore increased, due to the high surface volume ratio of the as-synthesized ZnO peanuts. It has been reported that the chemisorption of the picric acid molecules amends the electronic states of the ZnO nanomaterials and improves the conductance [45]. These phenomena, such as charge transfer, altered electronic states, and improved conductance can be attributed to the excellent sensing performances of the ZnO nanomaterials.

To ascertain the elemental composition, the synthesized ZnO nanopeanuts were examined by energy dispersive spectroscopy (EDS) attached with FESEM (Figure 1c,d). As confirmed from the EDS spectrum, the synthesized material is made of zinc and oxygen, as no other peak related with any other element is seen in the observed EDS spectrum. The presence of only zinc and oxygen peaks in the EDS spectrum confirmed that the synthesized nanopeanuts are pure ZnO without any significant impurity.

It has been reported that Zn^2+^ ions combine with ${\mathrm{NH}}_{4}^{+}$ and HO^−^ ions to form ${\left[\mathrm{Zn}{\left({\mathrm{NH}}_{3}\right)}_{4}\right]}^{2+}$ and ${\left[\mathrm{Zn}{\left(\mathrm{OH}\right)}_{4}\right]}^{2-}$ growth units in the reaction medium [46,47]. The detailed growth mechanism for ZnO nanopeanuts has been elaborated elsewhere [47].

**Figure 2:**
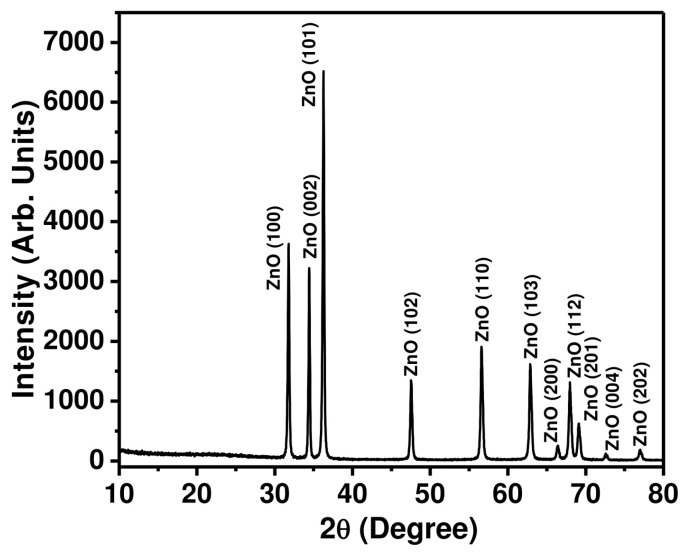
Typical XRD pattern for the as-synthesized ZnO nanopeanuts.

To further confirm the purity, crystal phases, and structure of the ZnO nanopeanuts, an X-ray diffraction pattern was recorded between 2θ = 10°–80°. Figure 2 represents the typical XRD pattern of the as-synthesized ZnO nanopeanuts, which indicates a hexagonal phase of pure ZnO in line with the reported literature [10,11,48,49]. The diffraction peaks observed at 31.6°, 34.3°, 36.3°, 47.4°, 56.6°, 62.8°, 66.5°, 68.5°, 69.7°, 74.3°, and 78.2° correspond to the lattice planes of ZnO (100), (002), (101), (102), (110), (103), (200), (112), (201), (004), and (202), respectively. No other diffraction peaks, except for a wurtzite hexagonal phase, are observed in the XRD pattern, which clearly confirmed that the synthesized nanopeanuts are only ZnO. The results of the XRD pattern matches that of the EDS observations.

**Figure 3:**
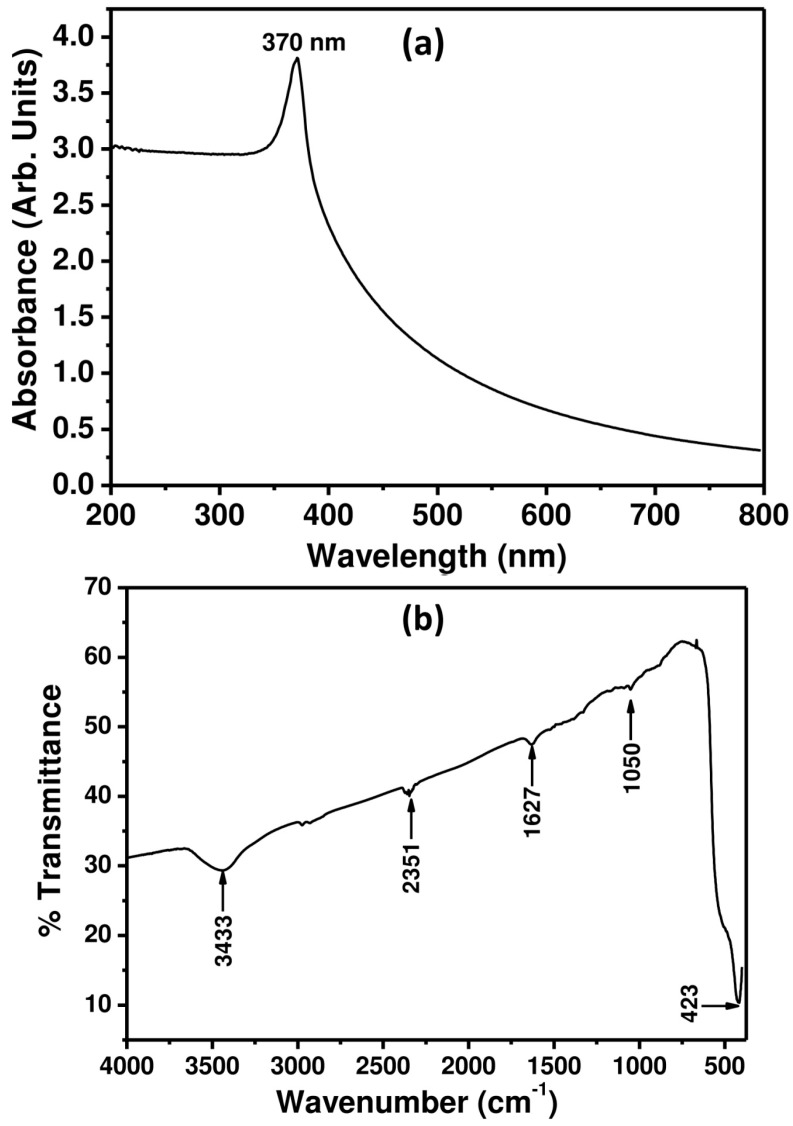
(**a**) UV-Vis and (**b**) FTIR spectra for the as-synthesized ZnO nanopeanuts.

The UV-Vis. absorption spectrum for the ZnO nanopeanuts is shown in Figure 3a. A well-defined single exciton absorption peak at 370 nm, corresponding to the pure wurtzite hexagonal phase, can be clearly seen, which is also in good agreement with the reported literature [48,49,50,51].

The band gap energy calculated using the well-known Planck’s equation (Equation (1)) was found to be 3.35 eV [52]. No other absorption peak, except for 370 nm, confirms the fact that ZnO nanopeanuts possess excellent optical properties.

(1) $$E_{g}=\frac{\mathrm{hc}}{\lambda_{\max}}=\frac{6.625\;\times 10^{-34}\mathrm{Js}\;.\;3\;\times 10^{8}{\mathrm{ms}}^{-1}}{370\times {\;10}^{-9}m.\;1.6\;\times {\;10}^{-19}\;J{.\;\mathrm{eV}}^{-1}}=3.35\mathrm{eV},$$

Figure 3b depicts a typical FTIR spectrum of the as-synthesized ZnO nanoflakes, which exhibits various well-defined bands appearing at 423, 1050, 1627, 2351, and 3433 cm^−1^. One sharp and well-defined peak at 423 cm^—1^ and another weak band at 1050 cm^−1^ may be assigned to the stretching and bending vibrational modes of the Zn-O bonds, respectively [49,53]. A weak band at 1627 cm^−1^ and a broad band 3433 cm^−1^ appear due to the bending and stretching vibration modes of the O–H groups, respectively, for the physisorbed water molecules on the surface of the ZnO nanopeanuts [52,54]. The low-intensity sharp band at 2351 cm^−1^ may be attributed to the asymmetric stretching of the C=O bonds of CO_2_ molecules, adsorbed from the environment during KBr palletization [54].

**Figure 4:**
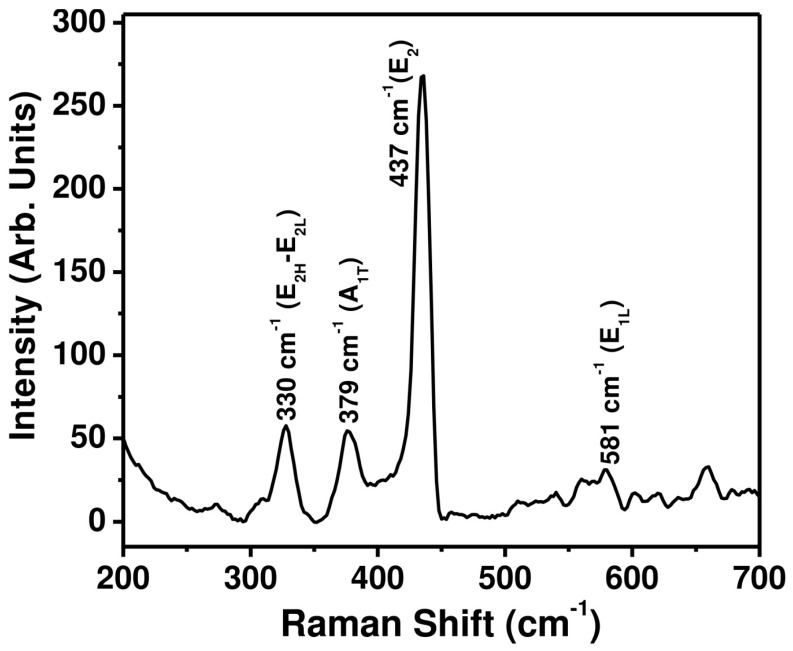
Typical Raman-scattering spectrum of the as-synthesized ZnO nanopeanuts.

To examine the scattering properties, the as-synthesized ZnO nanopeanuts were characterized by a Raman-scattering spectrum at room temperature, and the observed result is presented in Figure 4. The observed Raman-scattering spectrum exhibits various phonon peaks appearing at 330, 379, 437, and 581 cm^−1^, which is consistent with the reported Raman-scattering spectrum of ZnO nanomaterials [41]. ZnO with a wurtzite hexagonal phase belongs to the $C_{6v}^{4}$ (P63mc) space group having four ZnO units per primitive cell. The peak corresponding to the non-polar optical phonon appeared at 437 cm^−1^, and is assigned to the $E_{2}^{\mathrm{High}}$ mode. It is the characteristic peak for the wurtzite hexagonal phase of ZnO. The small but sharp peak at 330 cm^−1^ is assigned to the E_2H_–E_2L_ multi-phonon process. The other weak bands at 379 and 581 cm^−1^ are associated with the A_1T_ and E_1L_ modes, respectively. All of these peaks also match well with the reported literature values [55,56,57].

### 3.2. Performance of Fabricated Picric Acid Sensors Based on ZnO Nanopeanuts

#### 3.2.1. Sensing Properties of Picric Acid Sensor Based on ZnO Nanopeanuts Coated SPE

**Figure 5:**
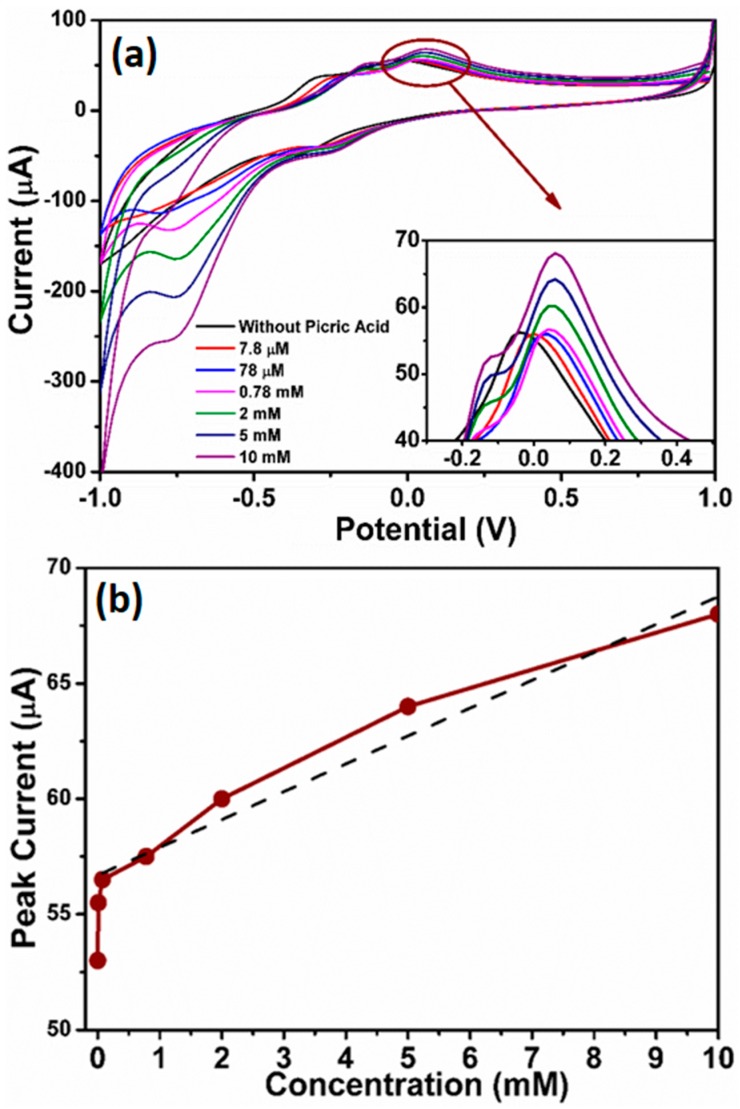
(**a**) Average cyclic voltammogram (CV) curves of the fabricated SPE at various concentrations of picric acid; and (**b**) Variation in the anodic peak current with concentration of picric acid.

Figure 5a shows the average CV curves of two sets of measurements of a fabricated SPE at various concentrations of picric acid (0.0078, 0.078, 0.78, 2, 5, and 10 mM) prepared in 0.1M PBS (pH = 7.4) solution. The curves were acquired at the scan rate of 50 mV/s by varying the potential from −1.0 to 1.0 V. The peaks observed in the CV loop are related to the oxidation and reduction of the analyte that occurred at the potential of 0.04 V and −0.74 V, respectively. A systematic increase in peak current is noticed when increasing the concentrations of picric acid, which can be clearly seen in the inset of Figure 5a. At lower concentrations of picric acid, the increase in peak currents at a lower concentration is comparatively lower than those at higher concentrations. A slight increase in peak potential is observed with increasing concentration due to changes in the dielectric constant, with analyte being used as an electrolyte. The increase in the peak current can be attributed to the increased ionic strength at the electrode-electrolyte interface, and hence the extent of the electro-catalytic reaction occurring at the surface of the modified electrode [58,59,60,61] (schematically shown later in Figure 9).

Figure 5b shows the monotonic variation in the anodic peak current with concentration, which clearly shows the sensor’s sensitivity to picric acid. A sudden increase in the peak current is noticed on addition of 7.8 µM picric acid to PBS, indicating that the developed device is able to detect change in the analyte concentration, and although the amount of change is less still a detectable change in peak current is noticed. It has been reported in the literature that electrochemical sensors are fairly sensitive to a level of pico-mole with a measureable peak current. A further increase of picric acid amount results in a systematic increase in the peak current, indicating a linear behavior of the developed sensor. This curve can be used as a calibration curve, and the sensitivity is therefore estimated as 0.12 µA/mM or 9.23 µA/mM·cm^−2^ in terms of per unit area of the working electrode. The lowest experimental limit is 7.8 µM, which is well below the safety limit (lethal dose, an indication of lethal toxicity of the material) of picric acid [62,63].

**Figure 6:**
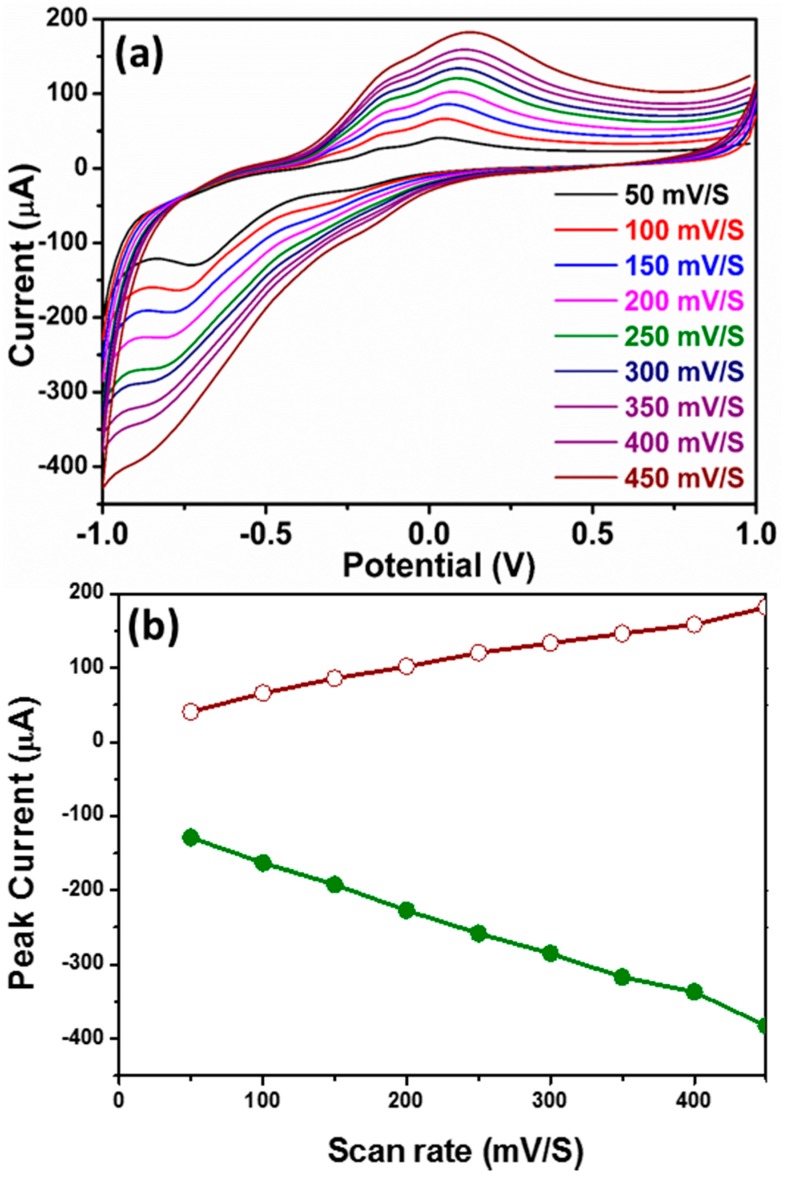
Typical (**a**) CV response curves at different scan rates (50, 100, 150, 200, 250, 300, 350, 400, and 450 mV/S) for a ZnO nanopeanut-modified SPE in 0.1 M PBS (pH = 7.4) containing 2 mM picric acid and (**b**) Variations of peak current with scan rate.

Further, for the effect of scan rate (kinetics) on the electrochemical properties of the synthesized material, scan rate dependent CV curves were obtained. Figure 6a shows the average CV curves obtained at scan rates such as 50, 100, 150, 200, 250, 300, 350, 400, and 450 mV·s^−1^ at a particular concentration of 2 mM picric acid solution. It is known that increasing the scan rate would result in increased diffusion/depletion resulting in increased peak current. Thus, from the increase in the peak currents for anodic as well as cathodic processes, it could be confirmed that the electron transfer process is a diffusion confined electrode process.

#### 3.2.2. Sensing Properties of Picric Acid Sensor Based on ZnO Nanopeanuts Coated on AgE

**Figure 7:**
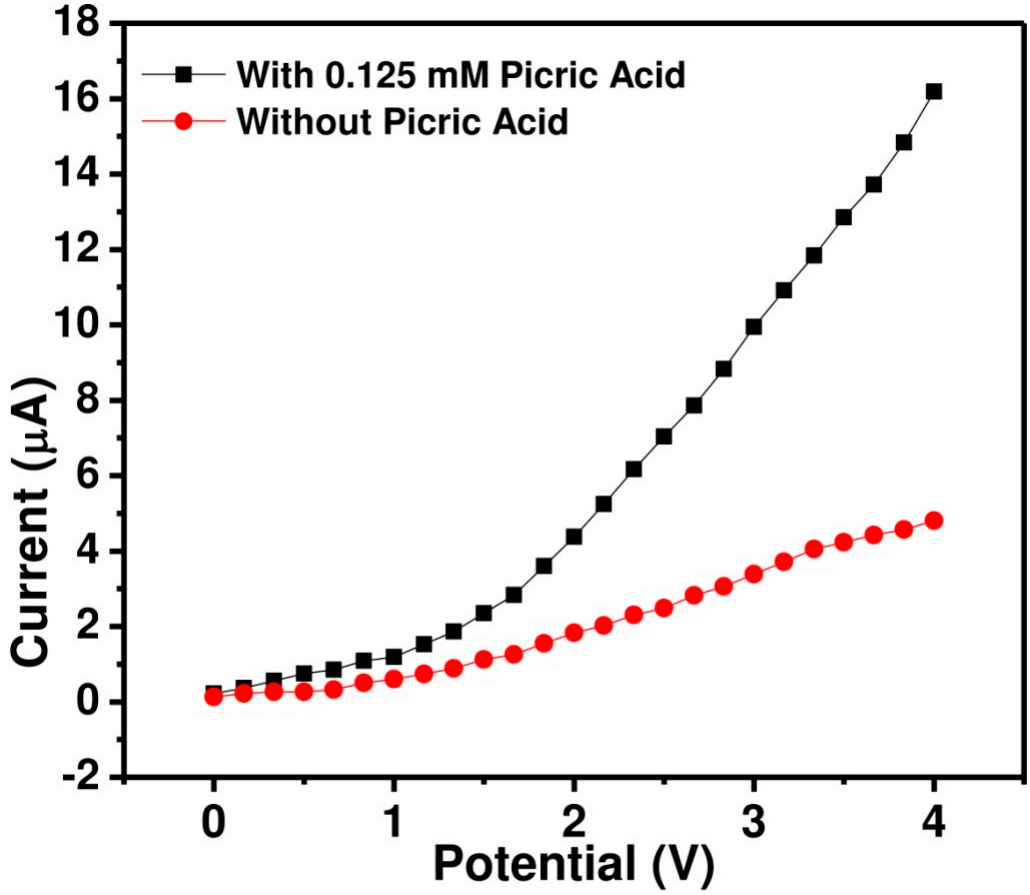
*I-V* performances for 0.125 mM picric acid and blank PBS using a ZnO nanopeanut-based AgE.

A ZnO nanopeanut based sensor was fabricated to evaluate the sensing characteristics of picric acid in 0.1 M PBS with pH = 7.4 by a simple current-voltage (*I-V*) technique. Figure 7 represents the Current-Voltage (*I-V*) responses for a blank 0.1 M PBS and 0.125 mM picric acid solution prepared in PBS having pH = 7.4, in the scan range of 0.0–4.0 V, and using ZnO nanopeanut-modified AgE and Pt wire as a counter electrode. Prominent current variations for the picric acid solution, as compared to blank PBS, indicates that the ZnO nanopeanut-modified AgE is involved in redox changes, and hence can be used as an efficient electron mediator for electrochemical sensor applications. At 4.0 V potential, a current response of 16.18 μA and 4.804 μA were observed for the picric acid and blank solutions, respectively.

**Figure 8:**
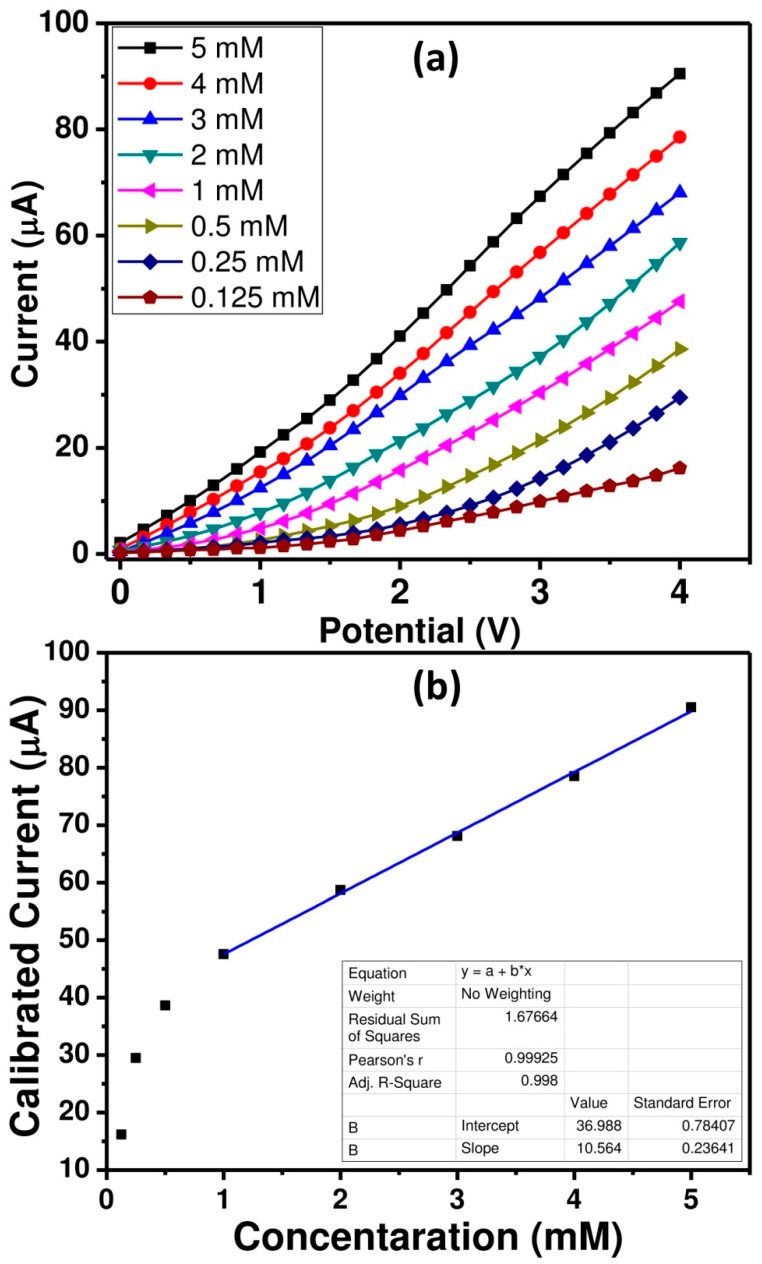
(**a**) *I-V* responses for various concentrations of picric acid solutions (**b**) Calibration graph for the fabricated ZnO nanopeanut-modified AgE electrochemical sensor.

Figure 8a depicts the effect of picric acid concentration on *I-V* responses. For 0.125, 0.25, 0.5, 1.0, 2.1, 3.0, 4.0, and 5.0 mM solutions of picric acid solution prepared in PBS, the corresponding current responses were 16.18, 29.48, 38.63, 47.58, 68.09, 78.53, and 90.49 μA, respectively, measured at 4.0 V. This positive correlation can be explained on the basis of an increase in the ionic strength of the PBS buffer solution with the concentration of the picric acid. Further, it can also be postulated that the greater the extent of chemisorptions of picric acid molecules at higher concentrations (through to a saturation point), the greater are the changes in the electronic states and conduction at the electrode-electrolyte interface of the *n*-type semiconducting ZnO nanopeanuts, and thus higher is the current response [60].

The sensitivity of the ZnO nanopeanut-modified AgE was calculated from the ratio of the slope of the calibration graph plotted between molar concentrations (0.125–5.0 mM) of the picric acid solutions and the corresponding current responses measured at 4.0 V, and the active surface area of the AgE (Equation (2)) [61] (Figure 8b).

(2) $$\mathrm{Sensitivity}=\frac{\mathrm{Slope}\;\mathrm{of}\;\mathrm{the}\;\mathrm{calibration}\;\mathrm{graph}}{\mathrm{Active}\;\mathrm{surface}\;\mathrm{area}\;\mathrm{of}\;\mathrm{the}\;\mathrm{modified}\;\mathrm{AgE}}$$

A very high sensitivity of 493.64 μA·mM^−1^·cm^−2^, an experimental LOD of 0.125 mM, a linear dynamic range (LDR) of 1.0 mM–5.0 mM and a regression coefficient of (*R*^2^) = 0.9980 were observed for the fabricated ZnO nanopeanut-modified AgE sensor. Due to the lower surface area of the electrode, a comparatively higher sensitivity is observed here than with the electrochemical sensing method. The sensitivity observed herein is high as compared to the reported sensitivities of some recently reported results. Huang et al. [64] reported a sensitivity of 0.00613 μA·μM^−1^ with a detection limit of 0.54 μM for picric acid through a reduced graphene oxide sensor modified with 1-pyrenebutyl-amino-β-cyclodextrin. It was proposed that β-cyclodextrin with the hydrophobic internal cavity and the hydrophilic external surface has a remarkable tendency to integrate with three hydrophobic -NO_2_ groups of picric acid. However, there is a a low limit of detection of 0.6 μM for a copper-based electrochemical sensor [65,66] and 100 nM for a boron and nitrogen co-doped carbon nanoparticles based photoluminescent sensor [67] as compared to the ZnO peanut-based electrochemical sensors in this study.

## 4. Proposed Sensing Mechanism

**Figure 9:**
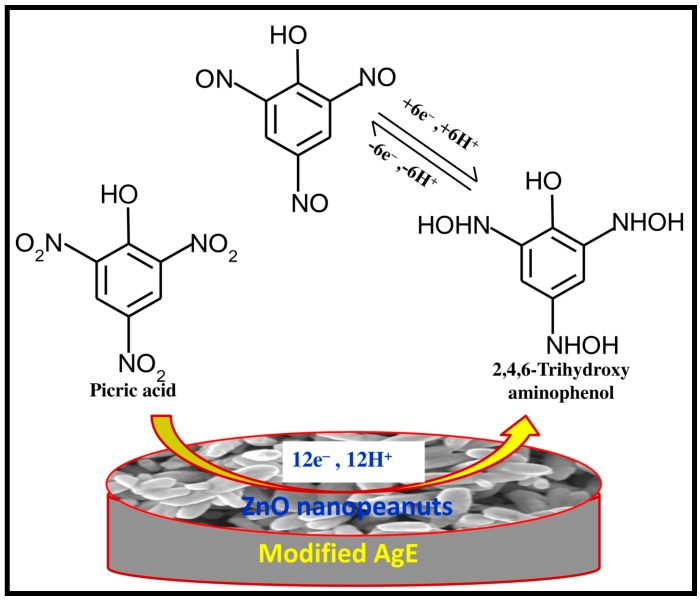
A sensing mechanism for picric acid sensing using modified AgE with ZnO nanopeanuts.

On the basis of Frontier molecular orbital studies, it has been shown that the adsorption of the picric acid molecules on the surface of ZnO lowers the energy gap between the highest occupied molecular orbital (HOMO) and the lowest unoccupied molecular orbital (LUMO), resulting in the alteration of the electronic states and hence the charge transfer and conductance of the ZnO nanomaterials, as stated earlier in Section 3.1 [14,45]. Picric acid molecules with electron donor hydroxy (–OH) and electron withdrawing nitro (–NO_2_) groups are adsorbed on the surface of the ZnO nanopeanuts through weak van der Waal interactions, where they undergo a series of redox changes [14]. Conduction band electrons of the ZnO nanopeanuts reduce the three –NO_2_ groups to intermediate hydroxylamino (–HN–OH) groups, which are subsequently oxidized to nitroso (–NO) groups (Figure 9). The –NO groups undergo reversible reduction to release electrons back to the conduction band of the ZnO nanopeanuts, which are responsible for the increases in conductivity and current response [68].

## 5. Conclusions

In summary, ZnO nanopeanuts were synthesized in a large quantity and characterized in detail, which revealed that the nanopeanuts possess high crystallinity and exhibit good optical and spectral properties. The synthesized nanopeanuts demonstrated high purity and were confirmed for the wurtzite hexagonal phase of pure ZnO. Further, the synthesized nanopeanuts were explored for their picric acid sensing applications in the form of an electrochemical and electrical sensor. Reliable, reproducible, and reversible CV and IV curves were obtained, indicating the versatility of the synthesized material for an efficient, sensitive, and reliable matrix for a picric acid sensor. Hence, ZnO nanopeanuts can be efficiently used as excellent electron mediators for the fabrication of sensors for other nitrophenols with very high sensitive and very low LOD.
